# Routine HIV Testing among Hospitalized Patients in Argentina. Is It Time for a Policy Change?

**DOI:** 10.1371/journal.pone.0069517

**Published:** 2013-07-31

**Authors:** María Eugenia Socías, Laura Hermida, Mariana Singman, Gisela Kulgis, Andrés Díaz Armas, Osvaldo Cando, Omar Sued, Héctor Pérez, Ricardo Hermes, José Luis Presas, Pedro Cahn

**Affiliations:** 1 Infectious Diseases Division, Hospital J. A. Fernández, Buenos Aires, Argentina; 2 Fundación Huésped, Buenos Aires, Argentina; 3 Internal Medicine Division, Hospital J. A. Fernández, Buenos Aires, Argentina; 4 Laboratory Division, Hospital J. A. Fernández, Buenos Aires, Argentina; Vanderbilt University, United States of America

## Abstract

**Introduction:**

The Argentinean AIDS Program estimates that 110,000 persons are living with HIV/AIDS in Argentina. Of those, approximately 40% are unaware of their status, and 30% are diagnosed in advanced stages of immunosuppression. Though studies show that universal HIV screening is cost-effective in settings with HIV prevalence greater than 0.1%, in Argentina, with the exception of antenatal care, HIV testing is always client-initiated.

**Objective:**

We performed a pilot study to assess the acceptability of a universal HIV screening program among inpatients of an urban public hospital in Buenos Aires.

**Methods:**

Over a six-month period, all eligible adult patients admitted to the internal medicine ward were offered HIV testing. Demographics, uptake rates, reasons for refusal and new HIV diagnoses were analyzed.

**Results:**

Of the 350 admissions during this period, 249 were eligible and subsequently enrolled. The enrolled population was relatively old compared to the general population, was balanced on gender, and did not report traditional high risk factors for HIV infection. Only 88 (39%) reported prior HIV testing. One hundred and ninety (76%) patients accepted HIV testing. In multivariable analysis only younger age (OR 1.02; 95%CI 1.003-1.05) was independently associated with test uptake. Three new HIV diagnoses were made (undiagnosed HIV prevalence: 1.58%); none belonged to a most-at-risk population.

**Conclusions:**

Our findings suggest that universal HIV screening in this setting is acceptable and potentially effective in identifying undiagnosed HIV-infected individuals. If confirmed in a larger study, our findings may inform changes in the Argentinean HIV testing policy.

## Introduction

Knowledge of HIV status is the gateway to HIV care and prevention services. Early HIV diagnosis and treatment has been shown to be beneficial to the individual patient by improving survival and quality-of-life [1–4], and in the broader public health perspective by decreasing HIV transmission [5–8]. Knowledge of HIV positive status has been associated with the reduction of unsafe sexual practices [9]. Moreover, timely initiation of highly active antiretroviral therapy (HAART) halts HIV replication, which delays disease progression and death, and decreases transmission of HIV through suppression of viral replication both in plasma and other fluids [10–13]. Therefore, there has been increasing interest in expanding the use of HAART as a tool to curb the growth of the HIV epidemic [14–19]. Identifying the majority of individuals living with HIV in a community has been proposed as the basis for effective use of HAART for prevention [20].

In Argentina, it is estimated that there are approximately 110,000 persons living with HIV/AIDS (PLWHA) with an estimated population prevalence of 0.4%; up to 40% are unaware of their status. Of those newly diagnosed in 2010, one-fourth were late diagnoses (according to the Argentinean AIDS Program definition: progression to AIDS within one year of HIV diagnosis) [21]. The Argentinean Public Health system guarantees free HIV testing for everyone living in the country and care, including antiretroviral drugs, for all eligible HIV positive persons. However, the high proportion of late diagnoses suggests that there are impediments to early testing, which may include socio-economic determinants and structural barriers that hamper access to testing and linkage to care.

The HIV epidemic in Argentina is concentrated both geographically and demographically. More than one-third of PLWHAs live in the city of Buenos Aires and its surrounding suburbs, and the estimated national prevalence of HIV is 12% in men who have sex with men (MSM), 7% in drug users, 2% in female sex workers, and 34% in transgender populations [21]. Additionally, the number of new HIV infections in populations not traditionally considered to be at risk is steadily growing [21].

Health institutions represent a key point of contact with HIV-infected individuals. However, research shows that many opportunities for early diagnosis and care are often missed, both in high- and low-income settings [22–24]. These missed opportunities translate into increased health care costs and continued transmission of HIV. Integrating routine HIV screening into clinical settings may help to overcome some of the barriers to testing.

Since 2006, the United States Centers for Disease Control and Prevention (CDC) has recommended routine HIV screening for all patients aged 13 to 64 presenting to healthcare facilities where the community HIV prevalence is greater than 0.1% [25]. Similarly, since 2008, United Kingdom guidelines recommend offering HIV testing to all general medical admissions or primary care registrants in areas where the diagnosed prevalence of HIV infection exceeds 0.2% [26].

In Argentina, with the exception of antenatal care and blood banks, HIV testing has always been client-initiated with the requirement of pre-test counseling and signed informed consent (opt-in approach).

In order to explore the potential impact of alternative strategies on expanding testing in our country we decided to offer HIV testing to all adults admitted to the internal medicine ward at an urban public hospital. The aims of this pilot study were to evaluate the acceptability of the program, and to estimate the undiagnosed HIV prevalence in this population and their demographic and clinical characteristics.

## Materials and Methods

### Ethics statement

The study protocol was approved by the J.A. Fernández Hospital institutional ethics committee.

In accord with the current Argentinean HIV legislation [27], written informed consent was required prior to HIV testing. Confidentiality was maintained by replacing patients’ names with study codes for HIV testing orders, HIV results, and questionnaires.

### Study Design

We undertook a prospective observational study between July and December 2011 to assess the acceptability of a pilot universal HIV screening program among inpatients at an urban public hospital from Buenos Aires city.

### Setting

Juan A. Fernández Hospital is an urban public tertiary hospital from Buenos Aires city, Argentina, with a catchment area that includes residents of the city, the surrounding suburbs, other provinces within Argentina, and persons from neighboring countries. The onsite Infectious Disease clinic has more than 4000 HIV positive patients on regular follow-up. We selected one inpatient internal medicine ward that admits approximately 60 patients per month to perform this study.

### Study population

All adults (older than 18 years old) who were admitted to the selected internal medicine ward over the study period and who were able to provide written informed consent were eligible for participating in the study. Only the first encounters were considered in patients who were admitted more than once during the study period. Exclusion criteria were self-reported HIV infection or inability to provide consent because of altered mental status or unstable medical illness.

### Pilot universal HIV screening program and observational study

Prior to initiating the pilot universal HIV screening program, two Internal Medicine attending physicians were trained in HIV testing issues, such as counseling, obtaining informed consent, and disclosure of test results. They were also specifically trained in completing the study questionnaires.

Within the first 48 hours of hospital admission, eligible patients received printed materials with information about HIV infection and testing and were invited to participate in the study. If they agreed, and after providing informed consent, one of the attending physicians administered a confidential one-page semi-structured questionnaire documenting the patient’s demographics, and HIV testing history, and offered the patient HIV testing free of charge. Demographic questions included sexual preferences, partnership, and substance use history. “Illicit drug/ substance use” was defined as a report of any of the following: history of injection drug use, use of marijuana, cocaine/“paco” (cocaine paste), LSD, ecstasy, or other recreational drugs. Once additional written consent for HIV testing was obtained (required by National Regulations) [27], a third generation HIV-1/2 ELISA was performed using a blood sample previously drawn for other laboratory tests. According to the standard protocol in Argentina, if the ELISA assay results were positive in duplicate, they were confirmed by Western blot. For patients who declined testing, reasons for refusal were assessed through open-ended questions and subsequently registered using a set of predefined codes. Responses that could not be codified in one of the available codes were included under the code “other”. Negative results were delivered by staff members with brief post-test counseling. Positive results (both preliminary and confirmed) were disclosed by an infectious diseases specialist who offered extensive post-test counseling and arranged a follow-up visit at the on-site HIV clinic before the patient’s discharge.

### Data management and statistical analysis

Data were entered in an anonymized excel database and analyzed using Stata/SE 11.1 (Stata Corp, College Station, Texas). The main outcomes assessed were: 1) number and proportion of eligible patients offered HIV tests, 2) number and proportion of patients who accepted HIV test, 3) number and proportion of persons with newly diagnosed HIV infections and their demographic and clinical characteristics, and 4) number and proportion of patients newly diagnosed with HIV infection successfully linked to HIV-care. Descriptive statistics for continuous variables were expressed as medians with interquartile ranges (IQRs) and categorical variables were presented as percentages. Comparative analyses were performed using Mann-Whitney and Fisher’s exact tests. Differences were considered statistically significant for p< 0.05, two-tailed tests. Logistic regression analysis was performed to examine factors that could be associated with the likelihood of HIV test uptake. Variables with p<0.1 in the univariable model were included in the multivariable model. Odds ratios (OR) and 95% confidence intervals (CI) were reported.

### ResultsHIV testing

**Figure 1:**
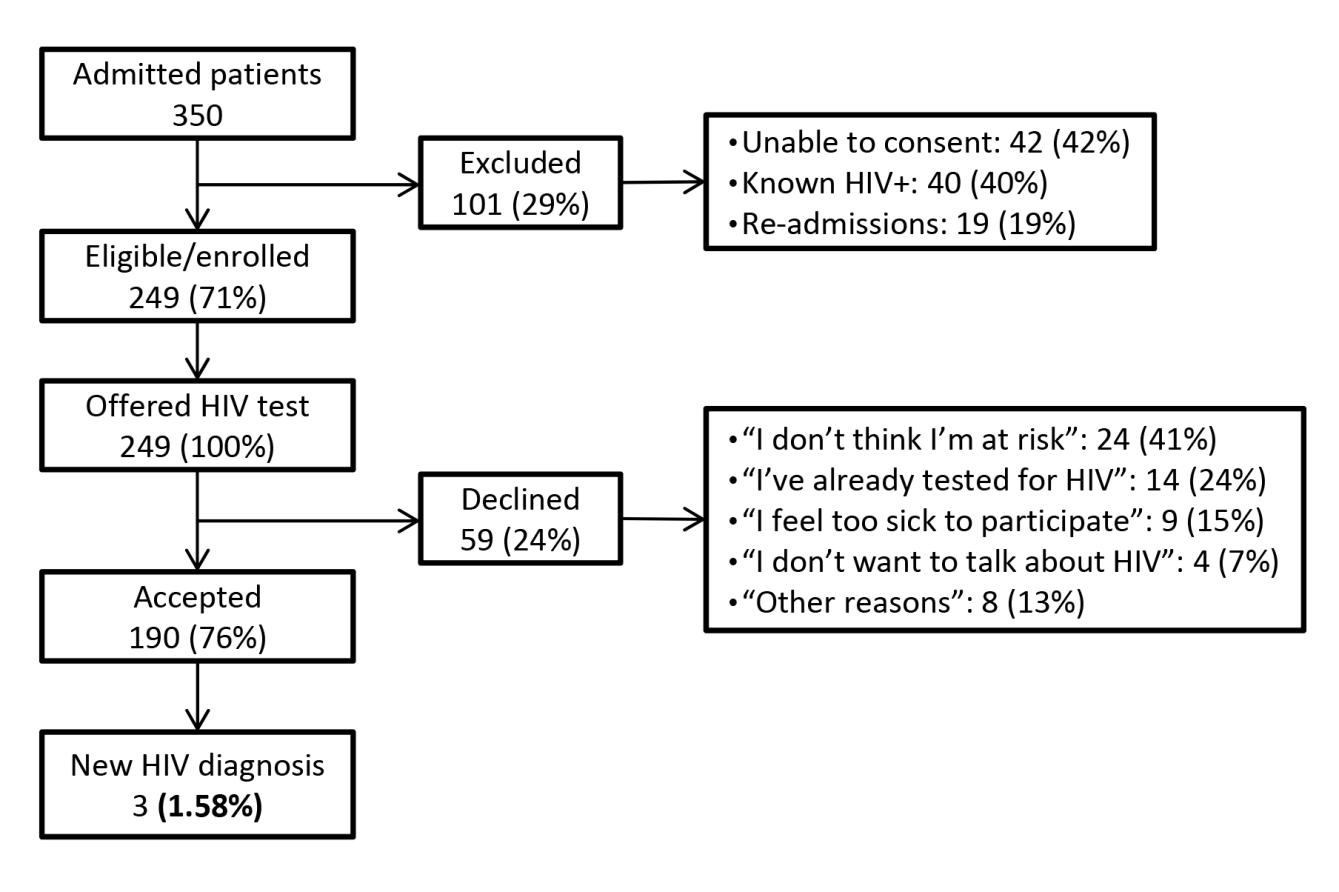
Patient flow diagram.

Between July and December 2011, there were 350 adult patient admissions to the selected internal medicine ward (Figure 1). Of these, 101 (29%) were excluded: 40 of them (39.6%) had a previous HIV diagnosis, 42 (41.5%) were unable to provide informed consent, and 19 (18.8%) corresponded to re-admissions during the study period.

All of the 249 eligible admissions (118 men and 131 women) agreed to participate in the study and were therefore enrolled. Of these, 190 consented to be tested for HIV (uptake rate 76.3%), while 59 declined. The most common reasons for refusal were not considering themselves at risk for HIV infection (41%), and having previously been tested for HIV (24%) (Figure 1). Other reasons for refusal were feeling too sick to participate in the study (15%), not wanting to talk about HIV (7%), and other causes (13%). Three new HIV diagnoses were made, corresponding to an undiagnosed HIV infection prevalence of 1.58% (95% CI, 0.19%–2.97%).

### Demographic characteristics of enrolled subjects

The majority of enrolled subjects were born in Argentina (78.3%). Overall, our study population was relatively old compared to the general population, with a median age of 58 years (IQR, 45–69), and had a balanced gender distribution (47% male). All participants reported being heterosexual and 21 (9%) reported use of illicit substances. None reported a history of intravenous drug use. Socio-economic status was low. Only 34% had completed high-school, 63.5% did not have medical insurance, and only 42% were currently employed. One hundred twenty-six patients (55%) were in a stable relationship, but only 12 (9.5%) of them were aware of their partner’s HIV status. Of note, just 39% reported having ever been tested for HIV before (Table 1).

**Table 1:** Demographic characteristics of enrolled patients (N=249).

	**Total N=249**	**Accepted HIV test n=190**	**Declined HIV test n=59**	**p**
**Argentinean, n (%)**	188 (78.3)	147 (77.4)	41 (82)	0.56
**Age in years, median (IQR)**	58 (45–69)	56 (42–65)	65 (49–76)	**0.004**
**Men, n (%)**	118 (47.4)	90 (47.4)	28 (47.5)	0.99
**Self-reported heterosexual, n (%)**	240 (100)	189 (100)	51 (100)	1
**Use of illicit drugs, n (%)**	21 (8.8)	19 (10.1)	2 (4)	0.26
**Completed high school, n (%)**	82 (34.3)	68 (36)	14 (28)	0.29
**Medical insurance, n (%)**	88 (36.5)	63 (33.2)	25 (49)	**0.04**
**Employed, n (%)**	102 (42.3)	83 (43.6)	19 (37.2)	0.41
**Stable relationship, n (%)**	126 (54.6)	97 (53)	29 (60.4)	0.36
**Previous HIV test, n (%)**	88 (38.9)	70 (38.9)	18 (39.1)	0.98

### HIV testing uptake

When we compared characteristics of patients who consented to HIV testing versus those who declined it, we found that the former group was on average 9 years younger (p=0.004) and less likely to have medical insurance (33% vs. 49%, p=0.04). We did not find statistically significant differences in any of the other studied variables, including gender (Table 1).

In univariable analysis test uptake was associated with younger age (OR 1.03; 95% CI 1.02–1.05) and not having medical insurance (OR 1.94; 95%CI 1.04-3.61). In the multivariable analysis, only age remained as an independent predictor of test acceptance (OR 1.02; 95% CI 1.003-1.05). Therefore, with each decrease in year of age, there was a 3% and a 2% increase in the odds of test uptake in univariable and multivariable analysis, respectively.

### Newly diagnosed HIV infection

Three patients were newly diagnosed with HIV infection, two men and one woman (Table 2). None of them belonged to a most-at risk population (MSM, IDUs, sex workers) and only one reported a previous negative test. Although only one of these three was admitted because of HIV-related symptoms, all three met national and international criteria for HAART initiation-even the most conservative ones [28–30]. Two patients were successfully linked to care, while the third was lost to follow-up.

**Table 2:** Characteristics of patients newly diagnosed with HIV infection (n=3).

**Gender**	**Age, years**	**Country of Birth**	**Previous HIV test**	**Transmission category**	**Use of drugs?**	**Hospitalization cause**	**HIV-related symptoms**	**CD4 cell count /μL**	**Linked to care?**
F	35	Argentina	No	Heterosexual	No	Interstitial pneumonia	Oral thrush	50	Yes
M	55	Paraguay	Yes (negative)	Heterosexual	No	Stroke	No	94	No
M	70	Brazil	No	Heterosexual	No	Intra-abdominal abscess	No	284	Yes

F: female; M: male

## Discussion

To our knowledge, this is the first published experience of a routine HIV testing program among inpatients in Latin America. Despite the requirement of informed consent as part of the current Argentinean opt-in testing policy [27], 76% of our study patients accepted HIV testing. This uptake rate is similar, or even higher, than uptake rates observed in studies done in similar clinical settings from the US [31–35], Europe [36–39] and Africa [40,41]. That all eligible patients agreed to participate in the study along with the high rate of HIV test uptake suggest that a large number of inpatients at our institution who might not otherwise ask for testing may be willing to receive HIV testing, and that universal screening in an internal medicine ward setting would be an acceptable intervention to patients.

One-fourth of enrolled subjects declined testing. In the adjusted analysis the only independent predictor of HIV test uptake was age, with younger patients being slightly more likely to agree (OR 1.02; 95% CI 1.003-1.05). Similar to other studies, the most common reason cited for declining HIV test was the lack of perception of being at-risk for HIV infection [36,39,42–45]. Other studies suggest that as a result of years of targeted screening programs for HIV, a perception has developed within the population that HIV testing is only necessary for those in certain “at-risk” groups [42,44,46]. However, as the demographics of the HIV epidemic have shifted, risk-based testing may be decreasing in effectiveness. This is consistent with HIV surveillance data [21,47–49], which indicate that late diagnosis remains a challenge worldwide, and that late testers are more likely to be older and to have been infected through heterosexual contact — groups not traditionally considered to be at high risk of HIV infection, and who might be missed in traditional targeted screening programs. A recent study in Buenos Aires city [50] found that out of 10,640 individuals newly diagnosed with HIV infection between 2003 and 2010, 37% were categorized as late diagnoses, defined as progression to AIDS or any other event associated with immunosuppression within one year of HIV diagnosis. This was more frequent among heterosexuals and people older than 40 years. This information strongly underscores the need of increased educational efforts to reverse the public perception that HIV testing should only offered to those considered “high-risk”.

More than 60% of our population had never been tested for HIV before. This could be due to lack of self-perceived risk or stigma, but it could also be that they had never been offered HIV testing from a health-care professional. A recent study in the United States found that although CDC’s recommendations for universal HIV screening have been in place since 2006, only 60% of health-providers are actually conducting routine HIV testing [51]. Universal offering of HIV testing to inpatients could represent an important opportunity to normalize HIV testing, identify individuals with undiagnosed HIV infection at an earlier stage of disease, and promptly link them to care.

Although enrolled subjects did not report traditional high-risk factors for HIV infection, three new HIV diagnoses were made. Two of these neither reported risk factors for HIV nor presented HIV-related symptoms, and would therefore probably have been missed if a targeted screening had been used.

The undiagnosed HIV infection prevalence of 1.6% found in our study is four times higher than the estimated population prevalence in Argentina [21]. This is above the 0.1% threshold at which the CDC recommends implementation of a universal HIV screening program at a health institution [25], and at which routine HIV screening is considered to be comparably cost-effective to the screening of other chronic and treatable diseases [48–50].

This study has a number of limitations. First, the study was conducted at a single, urban hospital medical ward in Buenos Aires city with a small sample size, and therefore our results may not be extrapolated to other areas within the hospital or other hospitals across our country. Second, our hospital hosts one of the main reference centers for HIV care in the country as reflected by the 40% of individuals who were excluded due to previous HIV diagnosis, and a selection bias for hospitalization of patients with HIV associated conditions cannot be ruled out. However, the diagnoses that prompted the hospitalizations were clearly not HIV-related in two out of the three cases. Finally, as information regarding risk factors for HIV infection and testing history was self-reported, social stigma could have influenced some patients’ responses and introduced bias in our analysis.

## Conclusions

In conclusion, to our knowledge this is the first study that explores the acceptability of universal HIV screening among inpatients in Argentina. We found that in our study setting, an inpatient internal medicine ward, this strategy was not only acceptable, but also effective in identifying individuals with undiagnosed HIV infection. This study should prompt the implementation of a larger scale study, and the exploration of this testing strategy in other settings, such as Emergency Departments.

This study was done without additional resources and relied on pre-existing staff who were highly motivated. Increasing workloads and staff shortages, both of which are common in Argentinean health institutions, may deleteriously affect the high screening rates observed in this pilot study, and these results may not be sustained over time. Therefore, streamlining pretest procedures, loosening current requirements for a separate written informed consent, or implementation of an opt-out approach may facilitate the inclusion of HIV testing into routine clinical care if a nationwide scale-up is initiated.

This manuscript has been presented in part at the 2012 HIV International TasP Workshop. Vancouver, BC, Canada. April 22–25, 2012
